# Exploring risk factors at the molecular level

**DOI:** 10.7554/eLife.68271

**Published:** 2021-04-13

**Authors:** Martina Rudnicki, Tara L Haas

**Affiliations:** Faculty of Health, York UniversityTorontoCanada

**Keywords:** aging, obesity, exercise, heart, endothelium, Human, Mouse

## Abstract

Risk factors for cardiovascular diseases trigger molecular changes that harm the endothelial cells in the heart, but exercise can suppress these effects.

**Related research article** Hemanthakumar KA, Fang S, Anisimov A, Mäyränpää MI, Mervaala E, Kivelä R. 2021. Cardiovascular disease risk factors induce mesenchymal features and senescence in mouse cardiac endothelial cells. *eLife* **10**:e62678. doi: 10.7554/eLife.62678

Being older, being overweight and having high blood pressure are all factors that increase the risk of developing cardiovascular diseases. Exercise protects against these diseases, but the details of how this happens at the cellular level are not fully understood.

The heart, like all other tissues, is composed of many types of cells, with endothelial cells – the building blocks for blood vessels – being one of the most common (Pinto et al., 2016). In the heart, endothelial cells control oxygen and nutrient supply, and contribute to immune protection (Aird, 2007). When endothelial cells malfunction, they affect neighboring cells and ultimately the health of the heart. Thus, it is recognized that endothelial cells have a central role in the development of cardiovascular diseases (Heitzer et al., 2001).

It is known that individual risk factors for cardiovascular diseases, such as obesity and aging, impair endothelial cell functions (Donato et al., 2007; de Jongh et al., 2004), but a number of questions remain unanswered. What are the effects of these risk factors at a molecular level? Do the different risk factors trigger a common set of disruptions in endothelial cells? And are the benefits of physical activity due to exercise repressing the detrimental molecular changes triggered by these risk factors?

Now, in eLife, Riikka Kivelä and colleagues at the Wihuri Research Institute and the University of Helsinki – including Karthik Hemanthakumar as first author – report that they have identified genes that are dysregulated by one or more cardiovascular risk factors and improved by exercise (Hemanthakumar et al., 2021). The researchers used mouse models that mimic human obesity, aging, cardiac overload and exercise training, and employed a procedure called fluorescence-activated cell sorting to collect cardiac endothelial cells. Hemanthakumar et al. then used RNA sequencing and various bioinformatics analyses to study the cells.

The analyses revealed that obesity and cardiac overload deteriorate the molecular characteristics of endothelial cells, in a similar way to aging (Figure 1). Cells from the mouse models for cardiac overload and obesity overexpressed genes associated with biological aging (senescence), inflammation, oxidative stress and pathways involving a signaling protein called TGF-β (which is involved in cell proliferation, differentiation and various other processes). Moreover, molecular pathways involved in cell number maintenance and vascular development were repressed by the same risk factors, indicating that these conditions impair the ability of the cells to coordinate the growth of new vessels. Obesity and aging also reduced the expression of genes traditionally associated with endothelial cell identity; instead, cells in these mice expressed genes associated with cells known as mesenchymal cells. This type of identity transition (endothelial-to-mesenchymal) has been linked to cardiovascular diseases in other studies (reviewed by Kovacic et al., 2019).

**Figure 1.**
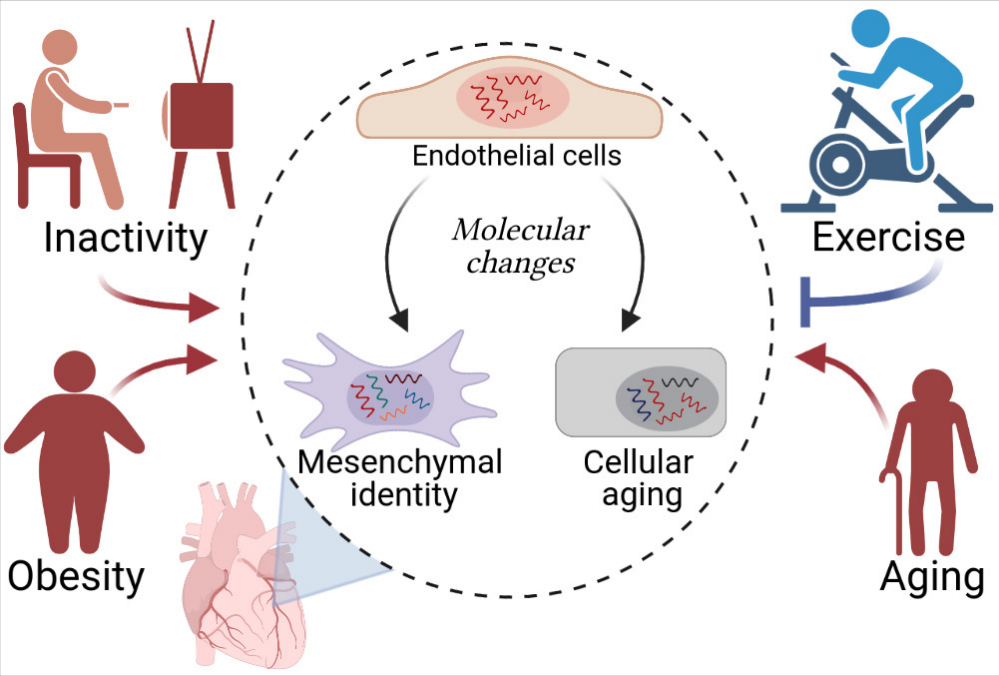
Cardiovascular diseases and cardiac endothelial cells. Hemanthakumar et al. show that risk factors for cardiovascular diseases (red) trigger molecular changes (red arrows) in cardiac endothelial cells (top cell in the central circle). This increases the expression levels of genes related to cellular aging (bottom right) and mesenchymal identity (bottom left). Exercise (blue) can protect against cardiovascular diseases by repressing these molecular changes (blue flat-ended arrow). Figure created using BioRender.com.

Exercise, on the other hand, suppressed the activity of genes associated with cellular aging and mesenchymal identity, and increased the expression of genes involved in endothelial development. In fact, the mice serving as a model for the effects of physical activity exhibited a higher density of blood vessels in their hearts.

Hemanthakumar et al. also identified a cluster of genes on which risk factors and exercise exert opposite effects. The levels of expression of one gene in this cluster (a gene called *Serpinh1*) were increased by aging and obesity, and decreased by exercise in both young and old mice. Experiments using human cells grown in vitro showed that expression of this gene can be driven by TGF-β and oxidative stress. Moreover, human endothelial cells that were forced to overexpress SERPINH1 became larger and expressed more genes related to senescence and mesenchymal-like identity.

While these results highlight the potential for targeting SERPINH1 to counteract the effects of various risk factors for cardiovascular diseases on endothelial cells, they also raise a number of questions. For example, it has been reported that the protein encoded by SERPINH1 – heat shock protein 47 – co-localizes with endothelial-to-mesenchymal markers in endothelial cells from patients with atrial fibrillation (Kato et al., 2017). But is the production of SERPINH1 increased in the cardiac endothelial cells of patients with cardiovascular diseases? If so, how does this increase contribute to cardiovascular diseases? Could blocking the endothelial production of SERPINH1 protect against the development of cardiovascular diseases associated with aging and obesity?

The study by Hemanthakumar et al. also reinforces existing evidence that physical activity improves the health of endothelial cells (Ashor et al., 2015). However, further studies are needed to explore which of the deleterious changes evoked by aging or obesity can be reversed by exercise.

Finally, what other genes should be explored to better understand cardiovascular pathology? In addition to SERPINH1, Hemanthakumar et al. have generated a wealth of information on genes dysregulated by major risk factors for cardiovascular diseases, and their findings could help to reveal additional endothelial genes that are important for cardiovascular health. This analysis will be a stepping stone towards a better understanding of the damaging effects of cardiovascular risk factors at the molecular level, paving the way to effective interventions.
